# Host Species and Environment Shape the Skin Microbiota of Mexican Axolotls

**DOI:** 10.1007/s00248-024-02411-1

**Published:** 2024-07-24

**Authors:** Enrique Soto-Cortés, Montserrat Marroquín-Rodríguez, Maria Delia Basanta, Yurixhi Maldonado-López, Gabriela Parra-Olea, Eria A. Rebollar

**Affiliations:** 1 Centro de Ciencias Genómicas, Universidad Nacional Autónoma de México, Cuernavaca, México; 2 Facultad de Medicina, Universidad Nacional Autónoma de México, Mexico City, México; 3 Department of Biology, University of Nevada Reno, Reno, NV USA; 4 Facultad de Ciencias, Universidad Nacional Autónoma de México, Mexico City, México; 5 Cátedras CONAHCYT - Instituto de Investigaciones Sobre los Recursos Naturales, Universidad Michoacana de San Nicolás de Hidalgo, Morelia, Michoacán México; 6 Instituto de Biología, Universidad Nacional Autónoma de México, Mexico City, México

**Keywords:** Amphibian skin, Axolotl, Microbiota, Paedomorphic salamanders, Ambystoma

## Abstract

Skin microbiomes in amphibians are complex systems that can be influenced by biotic and abiotic factors. In this study, we examined the effect of host species and environmental conditions on the skin bacterial and fungal microbiota of four obligate paedomorphic salamander species, commonly known as axolotls (*Ambystoma andersoni*, *A. dumerilii*, *A. mexicanum*, and *A. taylori*), all of them endemic to the Trans-Mexican Volcanic Belt*.* We found that despite their permanent aquatic lifestyle, these species present a host-specific skin microbiota that is distinct from aquatic communities. We identified skin-associated taxa that were unique to each host species and that differentiated axolotl species based on alpha and beta diversity metrics. Moreover, we identified a set of microbial taxa that were shared across hosts with high relative abundances across skin samples. Specifically, bacterial communities were dominated by Burkholderiales and Pseudomonadales bacterial orders and Capnodiales and Pleosporales fungal orders. Host species and environmental variables collectively explained more microbial composition variation in bacteria (R2 = 0.46) in comparison to fungi (R2 = 0.2). Our results contribute to a better understanding of the factors shaping the diversity and composition of skin microbial communities in *Ambystoma*. Additional studies are needed to disentangle the effects of specific host associated and environmental factors that could influence the skin microbiome of these endangered species.

**Supplementary Information:**

The online version contains supplementary material available at 10.1007/s00248-024-02411-1.

## Introduction

The skin of amphibians is a mucosal surface that is essential for many biological processes, including gas exchange, thermoregulation, osmoregulation, and defense [1]. It also harbors microbial communities, and some members of this microbiota are able to inhibit the growth of lethal pathogens, such as the chytrid fungus *Batrachochytrium dendrobatidis (Bd)* [2–4]. Thus, exploring the composition of amphibian skin microbial communities in a wide range of amphibian species and habitats may allow us to decipher specific microbial configurations that explain the protective role of skin microbiomes in amphibians [5].

Skin microbial communities in amphibians are dynamic and complex systems that are influenced by host-specific and climatic/environmental factors [6–8]. Host-associated traits like skin mucus chemistry, immunogenetic diversity, natural, and evolutionary history may drive selection for specific skin microbial assemblages in amphibians [7, 9]. Thus, host species identity can be an important predictor of skin microbial diversity [6, 10, 11]. In addition to host-associated factors, the amphibian skin microbiota is also influenced by the surrounding environment [12] due to the direct exposure of the host skin to external media [13]. It has been shown that microhabitat and local conditions influence local reservoirs of environmental microorganisms which in turn influence skin microbial composition [14]. Moreover, physicochemical components of the environment are factors that shape the amphibian skin microbiota [8, 15]. Particularly, it has been found that water pH and temperature have an influence on amphibian skin microbiota in aquatic habitats [16, 17]. In salamanders for instance, aquatic species have a distinct skin microbiota compared to terrestrial species [7]. Additionally, climatic and geographical factors at a larger scale, such as temperature, precipitation, seasonality regimes, and elevation, are associated to changes in amphibian skin microbial diversity and composition [7, 13, 18].

In this study, we explored the skin microbiota of aquatic salamanders from the genus *Ambystoma*. Species from this genus may exhibit facultative or obligate paedomorphosis. Paedomorphosis refers to the retention of juvenile characteristics in reproductive mature adult individuals [19]. Facultative paedomorphic species can metamorphose from aquatic larvae to terrestrial adults, whereas obligate paedomorphic species maintain an aquatic larval phenotype through their entire lives [20]. In Mexico, there are 17 *Ambystoma* species commonly known as axolotls [21], and there are only four species considered obligate paedomorphic salamanders: *Ambystoma andersoni*, *A. dumerilii*, *A. mexicanum*, and *A. taylori*. Each of these species is endemic to a single lake, all of them located along the Trans-Mexican Volcanic Belt. Their highly restricted distribution make these species extremely vulnerable to anthropogenic disturbances, climate change, and emerging diseases, and thus, all species are categorized as critically endangered [22].

We characterized the skin microbiota of these four axolotl species, which share life history traits (obligate paedomorphic) and habitat type (lakes). This will allow us to identify climatic and host-factors that may explain differences in skin microbial structure across host species. Specifically, we analyzed the diversity and structure of skin bacterial and fungal communities of the four species as well as their surrounding aquatic environment. We explored three hypotheses: (1) skin microbial diversity and structure will differ from their surrounding aquatic environment; (2) skin microbial diversity and structure will differ across host species; and (3) host species identity, climate, and habitat conditions will partially explain skin microbial composition. Our study is the first comparative assessment of the skin microbiota in obligate paedomorphic axolotls, thus contributing to a better understanding of the host-associated and environmental factors that could influence microbial communities in these threatened amphibian group of species.

## Methods

### Sampling Design and Collection

**Fig. 1:**
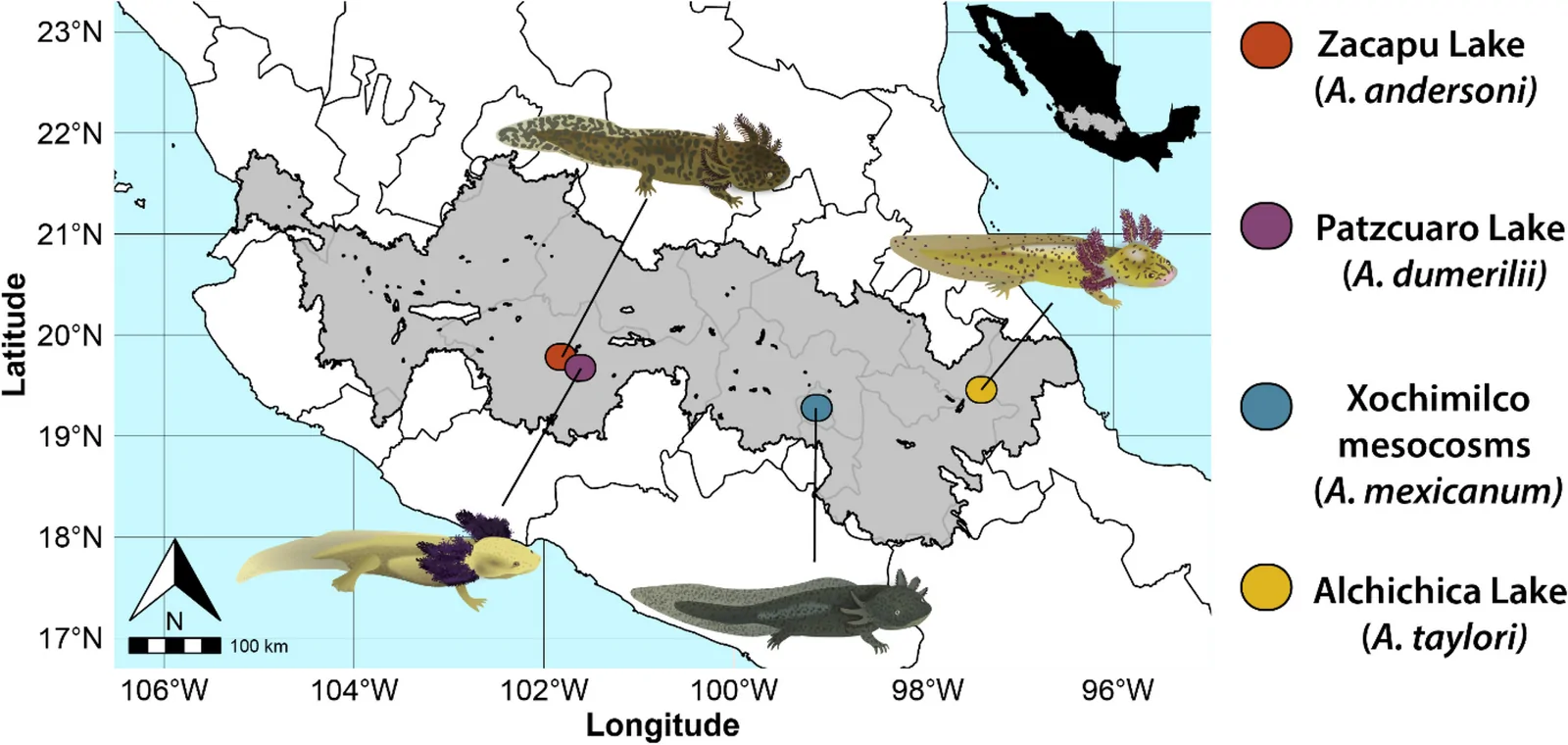
Sampling sites along the Trans Mexican Volcanic Belt, Mexico. The area in gray represents the Trans Mexican Volcanic Belt polygon. Each species distribution is restricted to a single lake: *Ambystoma andersoni* to Zacapu Lake; *A. dumerilii* to Patzcuaro Lake; *A. mexicanum* to a mesocosm system in Xochimilco; *A. taylori* to Alchichica Lake

We obtained a total of 93 skin swab samples from four *Ambystoma* species (*A. andersoni*, *A. dumerilii*, *A. mexicanum*, and *A. taylori*) between April and July of 2021 in four localities along the Trans Mexican Volcanic Belt (Fig. 1). *A. andersoni*, *A. dumerilii*, and *A. taylori* samples were obtained from their natural habitat, which are Lakes Zacapu, Patzcuaro, and Alchichica, respectively. *A. mexicanum* is a critically endangered species with a very small wild population, so we sampled this species from mesocosms of a captive colony from the Centro de Investigaciones Biológicas y Acuícolas de Cuemanco (CIBAC), UAM-Xochimilco. The mesocosms are under the same climatic conditions as the natural habitats and they use the Xochimilco Lake water as a source after it goes a filtration process to eliminate contaminants. To characterize the aquatic microbiota, a total of 26 water samples associated with each host habitat were obtained (Table 1). For further details in axolotl sampling, see Supplementary file 1.

**Table 1:** Sampling and location information of the four *Ambystoma* species analyzed in this study

Host species	Locality	Sampling period/date	Coordinates (latitude/longitude)	Habitat	Total skin samples	Total water samples	Total samples
*Ambystoma andersoni*	Zacapu, Michoacan	April–May 2021	19.823/ − 101.787	Lake	30	4	34
*Ambystoma dumerilii*	Patzcuaro, Michoacan	May–Oct 2021	19.675/ − 101.594	Lake	13	2	15
*Ambystoma mexicanum*	Xochimilco, Mexico City	April 23, 2021	19.281/ − 99.103	Mesocosm	27	15	42
*Ambystoma taylori*	Alchichica, Puebla	May 14–15 2021	19.406/ − 97.399	Lake	23	5	28
	Total				93	26	119

For skin microbial sampling, we rinsed each individual with 25 mL of sterile water before swabbing to reduce transitory microorganisms [23]. Following rinsing, we swabbed each individual 30 times (five times in ventral and dorsal surface and five times on each limb joint) with a sterile swab (MW-113, Medical Wire). Environmental water samples were collected with five sterile swabs at 20 cm deep for 10 s inside the water habitat of each location. Two negative control samples were taken for each location, one was a swab submerged in sterile water, and the second was a dry swab. All swab samples were placed in 1.5 mL sterile microcentrifuge tubes with 170 uL of DNA shield (Zymo Research, USA) and stored at 4 °C until arrival to the laboratory where tubes were stored at − 80 °C until processing.

### Bioinformatic Processing

DNA extraction, library construction, and sequencing methods are included in Supplementary file 1. Demultiplexed raw reads were pre-processed and filtered using Quantitative Insights Into Microbial Ecology (QIIME2,v. 2021.2) [24]. Sequences were quality-filtered and denoised using the DADA2 plugin, and then, sequences were clustered into representative sequences, known as amplicon sequence variants (ASVs). Taxonomy was assigned using a pre-trained naive Bayesian classifier which, for the bacterial dataset, was the V4 region using the SILVA 132 99% database [25], and for the fungal dataset was the ITS1 region on the UNITE database [26].

Two sets of QIIME2 artifacts “feature-table.qza” and “taxonomy.qza” were generated, one from 16S data and one from ITS data. Each dataset was imported into the R environment as a phyloseq object [27, 28] with the R package qiime2R [29]. Bacterial and fungal ASVs classified as chloroplast, mitochondria, archaea, eukaryota, and unclassified reads at the Phylum level were removed. After visual inspection of rarefaction curves, bacterial and fungal samples were rarefied at 10,000 reads per sample to normalize read counts. After rarefaction, one bacterial sample and 14 fungal samples were removed.

### Skin Microbial Composition Analyses

We defined an extended core microbiota (from now on “skin-associated microbiota”) to identify the ASVs that were likely more associated with the host skin than with the aquatic environment. The skin-associated microbiota was obtained for each host species considering all ASVs that had a total abundance > 0.001% of all reads and considering the following inclusion criteria: (1) all ASVs that were unique only to the skin samples (not present in water samples); (2) all ASVs present in at least 80% [30, 31] of skin samples, excluding ASVs that were highly prevalent in water samples; (3) all ASVs that were significantly enriched on the skin using DESeq2 [32] compared with water samples. The ASVs that were identified in one or more of the three former criteria (Figure S4) were used to generate a phyloseq file for each species and a single phyloseq file including the skin-associated microbiota for all species. This was done for the bacterial and fungal datasets independently. In the end, we generated four datasets for further analyses: “all.data.bac” and “all.data.fungi” which included all skin and water samples for bacteria and fungi, respectively. Datasets “core.data.bac” and “core.data.fungi” included the ASVs identified as part of the skin-associated microbiota for bacteria and fungi, respectively. All Venn diagrams were generated with the R package ggvenn [33].

To explore the skin-associated microbiota composition, we used the datasets “core.data.bac” and “core.data.fungi.” We visualized the shared and unique ASVs among host species with Venn diagrams [33]. Community taxonomic composition was visualized with stacked bar plots including the most abundant taxa at the order level using the microbiome package [34]. To determine which ASVs were differentially enriched on each host species, we performed linear discriminant analyses of effect sizes using LEfSe using *microbiomeMarker* package [35]. We ran a linear discriminant analysis (LDA) considering LDA scores > 3 for bacterial and fungal data [36, 37].

### Microbial Diversity Analyses

All diversity analyses, statistical tests, and graphical representations were performed in R v. 4.1.1 [27]. We evaluated differences in microbial community diversity between water and skin samples (using datasets “all.data.bac” and “all.data.fungi”). Then, we evaluated differences in skin microbiota alpha and beta diversity among host species (using datasets “core.data.bac” and “core.data.fungi”).

We used the function *alpha* from *microbiome* package [34] to calculate observed ASVs as an alpha diversity metric. We applied Shapiro normality tests. Since our data was not normally distributed, we used non-parametric tests: Wilcoxon test to determine differences in alpha diversity between water and skin samples, Kruskal–Wallis test to determine differences among host species, and Dunn’s test to determine pairwise differences between host species. Beta diversity was calculated using Bray–Curtis and Jaccard distance matrices and visualized in principal coordinate analyses (PCoA) using the *phyloseq* package [28]. To evaluate differences in beta diversity between host species and water, we used the *adonis* function within the *vegan* package [38] and performed a permutational analysis of variance (PERMANOVA) with 999 permutations. Beta diversity dispersion was calculated from the Bray–Curtis and Jaccard distance matrices using the function betadisper in the vegan package [38], followed by a PERMUTEST with 999 permutations. Specifically, we evaluated dispersion between skin and water communities, as well between host species microbial communities.

### Predictors of Alpha and Beta Diversity

We first used bioclimatic, physicochemical, and elevation data to describe environmental differences among sampling sites by performing a principal component analysis (PCA) and a PERMANOVA test. Then, to evaluate the effect of different factors on skin microbial diversity (using datasets “core.data.bac” and “core.data.fungi”), we used a metadata matrix (Table S8) considering the abiotic (bioclimatic, physicochemical, and elevation data) and biotic (body size and weight) variables to implement a two-step approach selecting the variables that remained as predictors of microbial alpha diversity: (1) pairwise Pearson correlations among selected variables to identify and discard those with a pairwise correlation higher than *r* > 0.7; (2) the least-correlated variables, together with host species, were included in a stepwise forward and backward regression model to select variables with significant effects on observed ASVs using the function stepAIC in MASS [39]. We implemented this approach for bacterial and fungal observed ASVs, separately. Before variable selection, we standardized the data with the function decostand (“standardize”) in the *vegan* package [38]. The selected variables were included to fit the linear model as fixed predictors using the R stats package [27]. The first model (bacteria) included the following variables: temperature annual range (Bio_7), precipitation (Pp), and monthly min temperature (Tmin) as predictors. The second model (fungi) included water temperature, Bio_7, min temperature of coldest month (Bio_6), and Pp as predictors.

To evaluate the effect of biotic and abiotic factors on beta diversity, we implemented a distance-based redundancy analysis (dbRDA) on the bacterial and fungal Bray-Cutis distance matrices using the capscale function from the *vegan* package [38]. We selected the least-correlated variables, together with host species as previously described, then we used the function ordistep from the *vegan* package [38] to select the best dbRDA models. We also included in the models the variables with a variance inflation factor (VIF) < 10. After variable selection, the dbRDA model for bacteria included water pH, water temperature, Tmin, Pp, and host species. The model for fungi included the same variables with the exception of Tmin. We employed a PERMANOVA to test the effect of individual predictor variables on beta diversity (anova.cca, by = terms).

## Results

### Axolotl Skin Microbial Structure Differs from Its Aquatic Environment

After quality filtering and rarefaction of bacterial and fungal datasets, a total of 118 bacterial and 105 fungal samples remained for further analyses, including samples from the skin of four axolotl species and water from the four habitats (Table S2). A total of 11,023 and 2753 amplicon sequence variants (ASVs) were obtained for bacteria and fungi, respectively.

**Fig. 2:**
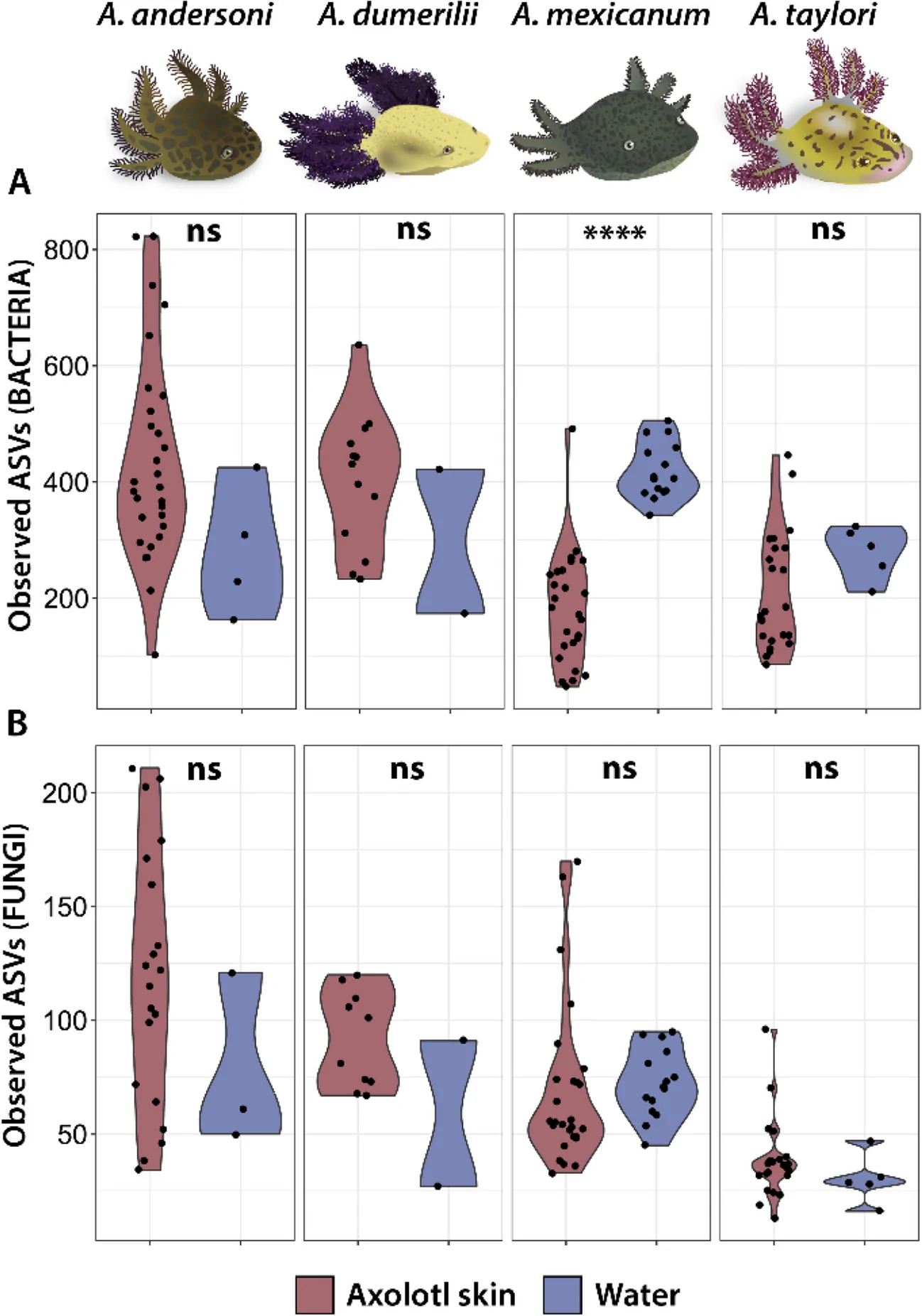
Microbial alpha diversity differences between axolotl skin and water samples for each host species and their respective habitats. **A** Observed bacterial ASVs and **B** observed fungal ASVs. Colors in the bottom legend denote the sample type. The asterisks above the bars indicate statistically significant differences between sample types: **** = *p*-value < 0.0001 and ns = non-significant

We found significant differences in bacterial alpha diversity (observed ASVs) between all skin and water samples, with water samples having lower diversity than skin samples (Wilcoxon, W = 812.5, *p* = 0.013). Moreover, we made comparisons between each host species and their respective water samples and found that only in *A. mexicanum* (Wilcoxon, W = 14, *p* < 0.001) skin samples were significantly different than water samples (Fig. 2A). In the case of the fungal community, we did not find significant differences in observed ASVs between all skin and water samples (Wilcoxon, W = 1056, *p* = 0.68) nor between each host species and its associated water samples (Fig. 2B). We identified that the majority of the bacterial and fungal ASVs were unique to the skin of each host species. However, in *A. mexicanum*, a larger proportion of bacterial and fungal ASVs were also shared with the water samples (Figure S1 and Figure S2).

**Fig. 3:**
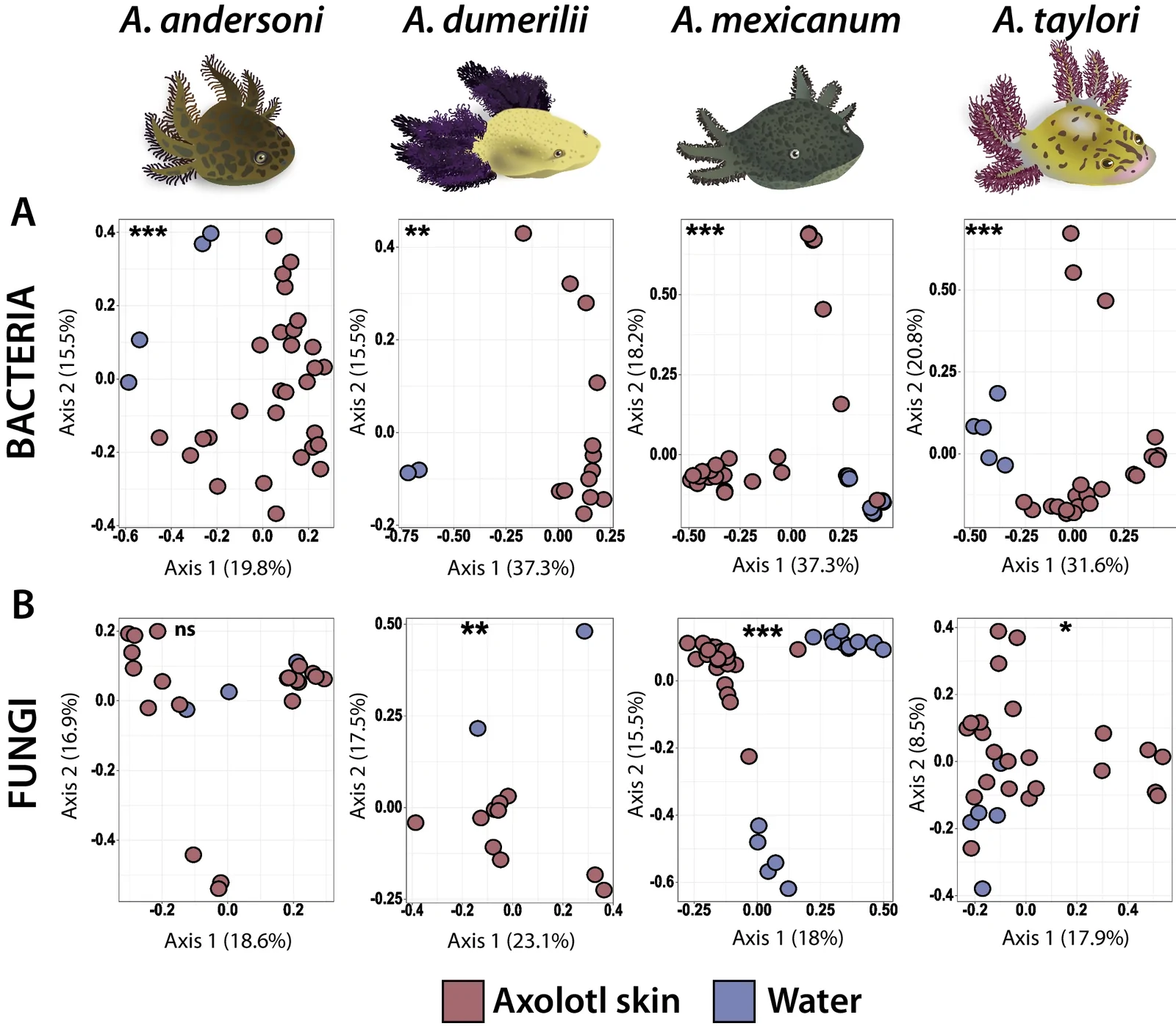
Beta diversity (Bray–Curtis dissimilarity) of axolotl skin and water microbiota for each host species and its respective habitat. Principal coordinates analysis (PCoAs) of (**A**) bacterial and (**B**) fungal communities of *Ambystoma andersoni*, *A. dumerilii*, *A. mexicanum*, and *A. taylori* (ordered left to right). Colors in the bottom legend denote the sample type. Asterisks indicate statistically significant differences between sample types: * = *p*-value < 0.05, ** = *p*-value < 0.01, *** = *p*-value < 0.001, ns = non-significant

Beta diversity analyses showed significant differences between skin and water communities based on Bray–Curtis and Jaccard distances, for both bacteria (PERMANOVA, Bray–Curtis: Pseudo-F = 10.32, *p* < 0.001; Jaccard: Pseudo-F = 4.37, *p* < 0.001) and fungi (PERMANOVA, Bray–Curtis: Pseudo-F = 4.69, *p* < 0.001; Jaccard: Pseudo-F = 1.95, *p* < 0.001). Moreover, we identified that skin microbial structure of each host species was more similar to their corresponding aquatic microbial communities than to other hosts (Figure S3). Thus, we decided to compare samples from each host with their associated water samples separately (Fig. 3). Based on Bray–Curtis distances, we found significant differences for all pairwise comparisons (water vs skin) except for the fungal communities in *A. andersoni*. When we considered Jaccard distances, all pairwise comparisons were significantly different for bacteria and fungi (Table S3 and Table S4).

We also compared the dispersion between water and skin samples. With Bray–Curtis distances, we found significant differences in dispersion between sample types for *A. andersoni* and *A. mexicanum* in the case of bacterial communities and for *A. taylori* in the case of fungal communities. With Jaccard distances, we found that dispersion was significantly different between sample types when all samples were considered and for all pairwise comparisons (water vs skin) per axolotl species (Table S3 and Table S4).

### Skin Microbial Diversity and Composition Differs Across Host Species But a Group of Abundant Taxa Are Shared Across All Axolotl Species

**Fig. 4:**
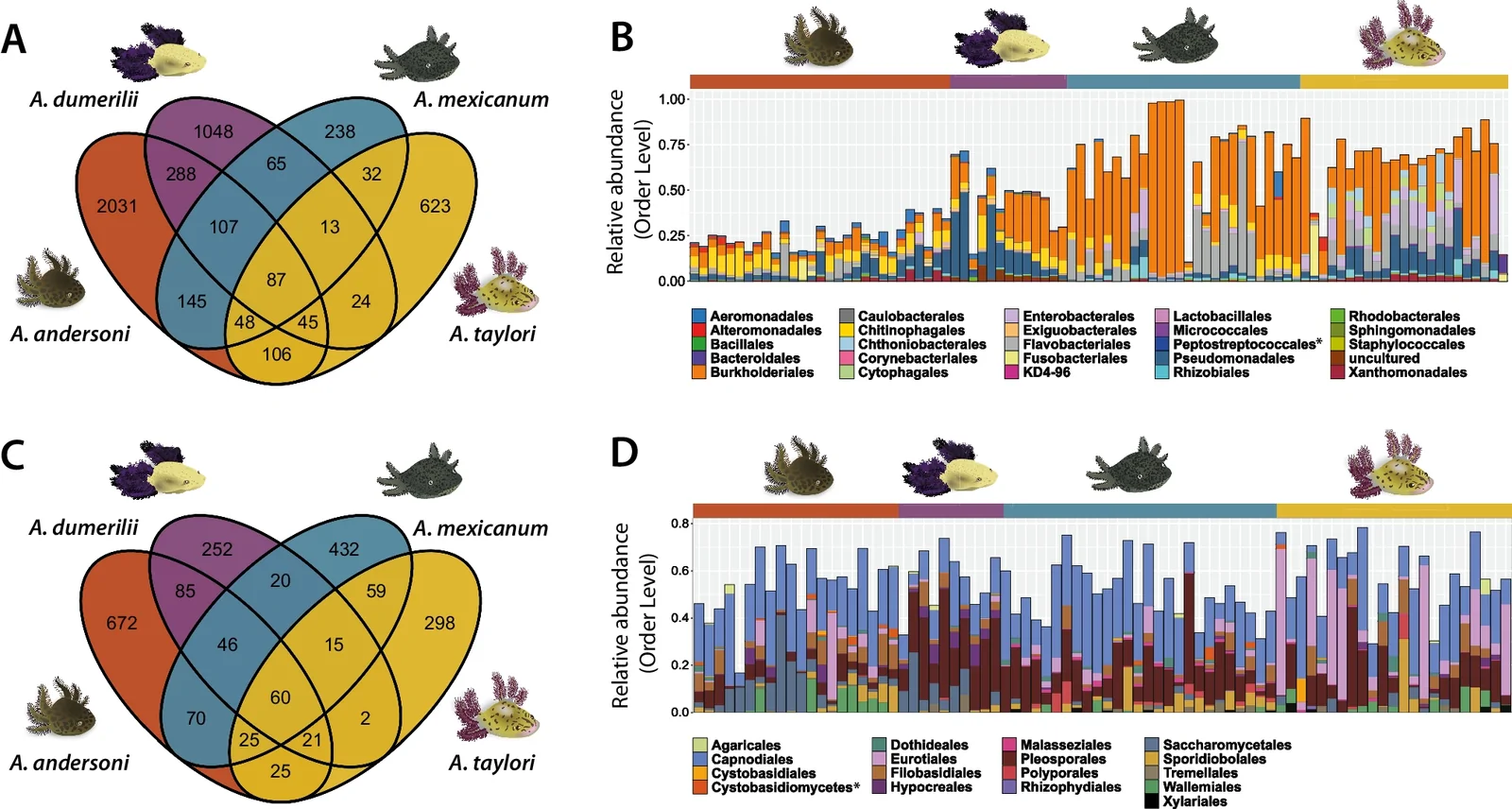
Composition of the skin-associated microbiota in axolotls. Venn diagram showing unique and shared **(A)** bacterial and (C) fungal ASVs among host species. Relative abundance at the order level of (B) bacterial and (D) fungal ASVs that are shared across all host species. Asterisks in taxonomic classification legends indicate names that have been abbreviated; for full names, see Table S9

We defined a set of skin-associated microbiota for each host species through identifying unique, enriched, or prevalent ASVs on the host skin and removing ASVs that are likely transitory taxa coming from water samples (see selection criteria in the “Methods” section). Skin-associated bacteria included 2857 ASVs in *A. andersoni*, 1677 ASVs in *A. dumerilii*, 735 ASVs in *A. mexicanum*, and 978 ASVs in *A. taylori*. Skin-associated fungi included of 1004 ASVs in *A. andersoni*, 501 ASVs in *A. dumerilii*, 727 ASVs in *A. mexicanum*, and 505 ASVs in *A. taylori*. When comparing the skin-associated microbiota among host species, we found that most of the ASVs were unique to each species (Fig. 4A and Fig. 4C). However, we were able to identify 87 bacterial ASVs and 60 fungal ASVs that were shared across all host species (Table S9). The shared, most abundant bacterial ASVs were classified as part of the Burkholderiales, Pseudomonadales, Chitinophagales, and Flavobacteriales orders (Fig. 4B), and they each had a mean relative abundance of 39.8%, 15.6%, 11.7%, and 10.5%, respectively, representing 77.6% of the mean total relative abundance in all axolotl species. On the other hand, the shared most abundant fungal ASVs were classified as part of the Capnodiales, Pleosporales, Eurotiales, and Saccharomycetales orders (Fig. 4D), and they each had a mean relative abundance of 37.3%, 23.8%, 9.3%, and 8.5%, respectively, representing 78.9% of the mean total relative abundance in all axolotl species.

**Fig. 5:**
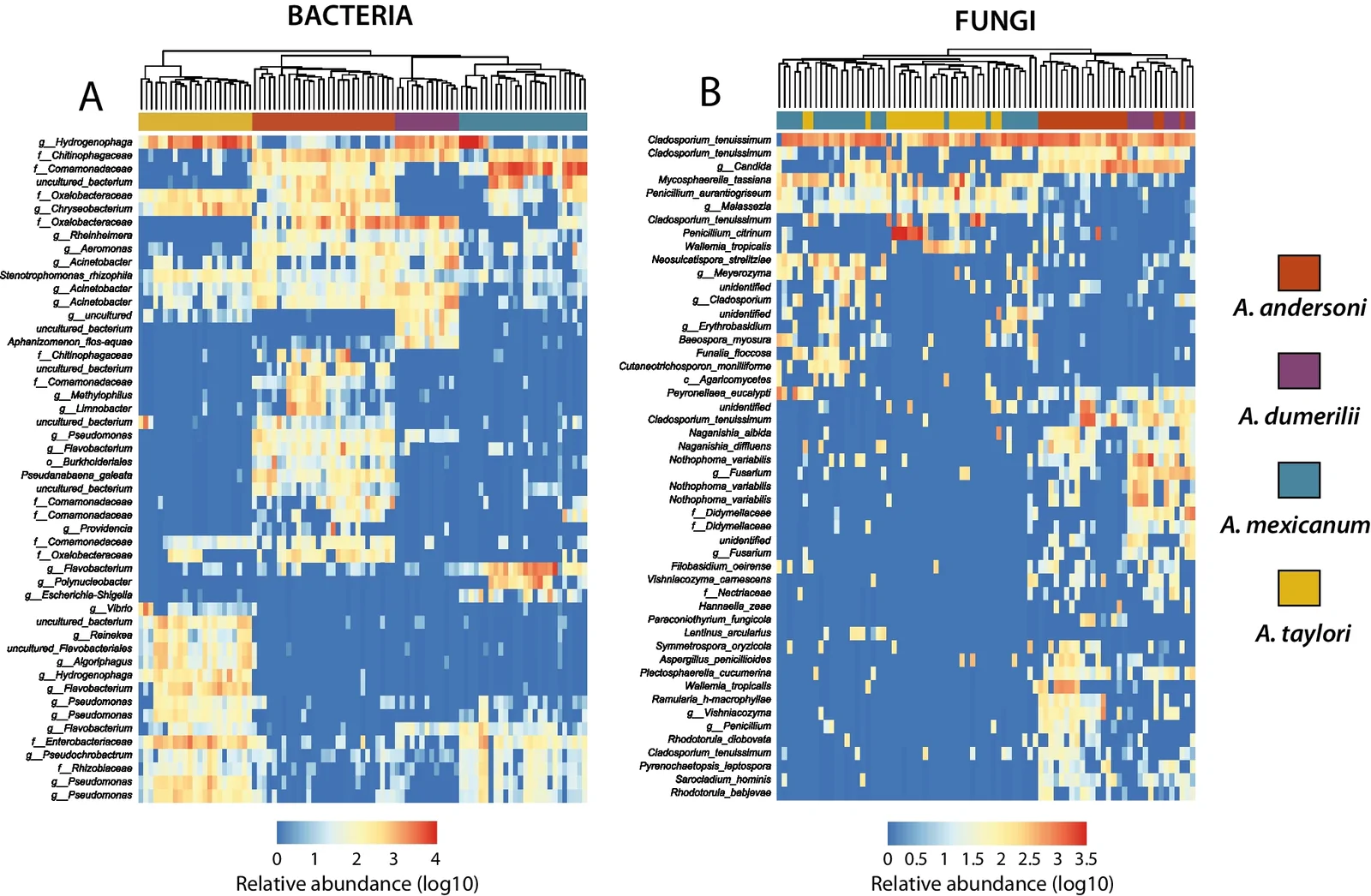
Heatmap showing relative abundance of bacterial and fungal ASVs that were significantly differentiated across host species using LEfSe analysis. The heatmap shows the 50 most abundant taxa for visualization purposes. Color gradient of the heatmap indicates log10 transformed relative abundances. Each column represents an individual sample, and each row represents a single ASV. Host species are indicated in the right legend with different colors. The dendrogram at the top was constructed with Bray–Curtis distances

To identify bacterial and fungal ASVs whose relative abundances explained differences among host species, we implemented a linear discriminant analysis effect size (LefSe, LDA score > 3). We found 148 bacterial and 85 fungal ASVs with statistically significant differences among host species (Table S10). The ASVs differentiating host species were 51 bacteria and 35 fungi for *A. andersoni*, 45 bacteria and 27 fungi for *A. dumerilii*, 23 bacteria and 19 fungi for *A. mexicanum*, and 29 bacterial and 4 fungal *A. taylori* (Fig. 5). Clustering samples (Bray–Curtis distances) based on LefSe results indicated that bacterial ASVs were clearly grouped by species (Fig. 5A) while fungal ASVs were clustered in two groups: *A. mexicanum-A. taylori* and *A. andersoni-A. dumerilii* (Fig. 5B).

**Fig. 6:**
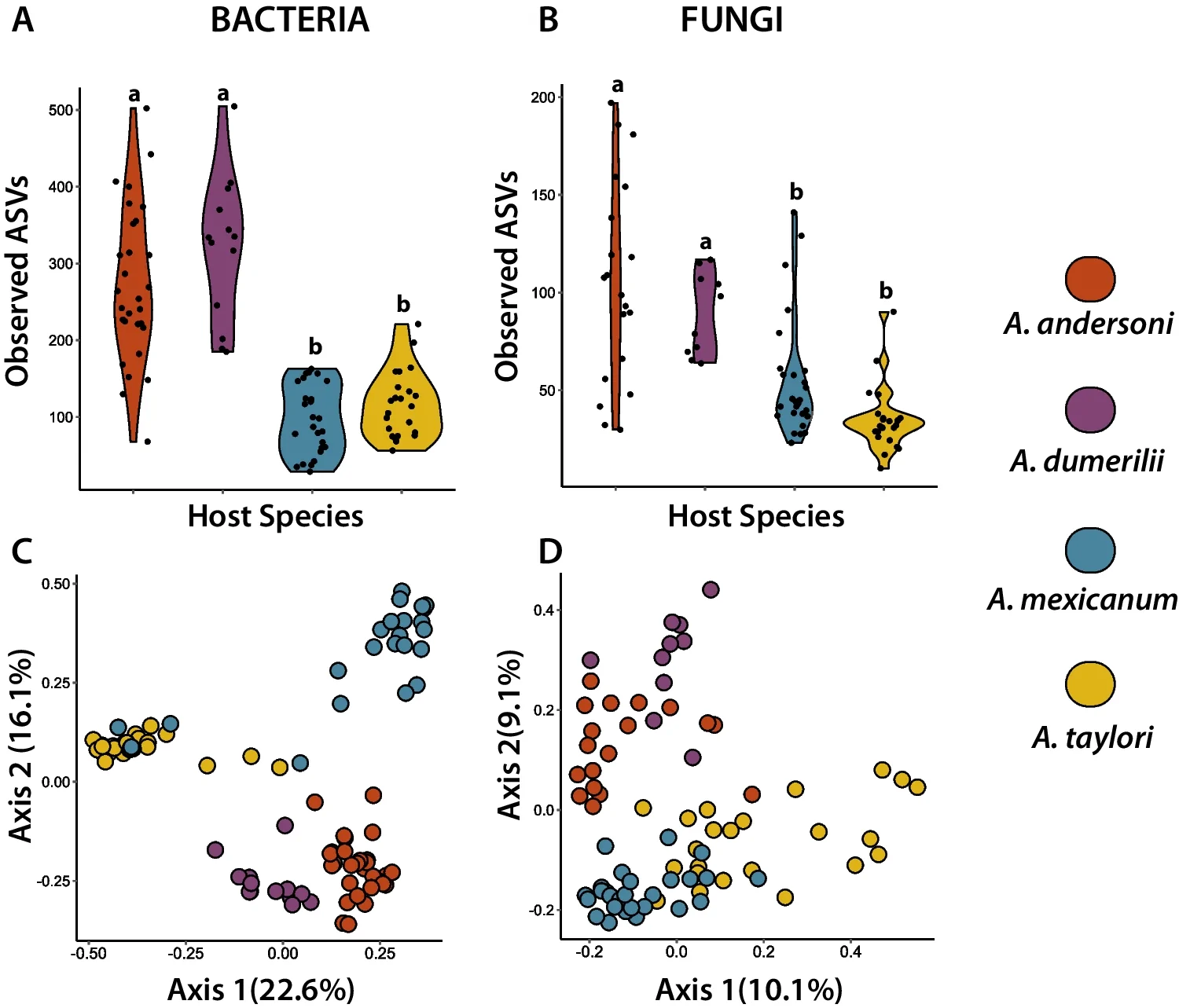
Alpha diversity levels (observed ASVs) of skin-associated microbiota among axolotl species for (A) bacteria and (B) fungi. Beta diversity (Bray–Curtis dissimilarities) of skin microbiota among axolotl species shown as a principal coordinates analysis (PCoAs) for (C) bacteria and (D) fungi. Letters in violin plots indicate significant differences among host species using post hoc Dunn’s test. Each color represents a different host species as shown in the legend

To further evaluate differences on skin microbial alpha and beta diversity among hosts species, we calculated observed ASVs and Bray–Curtis/Jaccard distances, respectively. We found significant differences in observed ASVs among host species for both bacterial (Kruskal–Wallis (KW), *X*^2^ = 58.4, *p* < 0.001) and fungal skin-associated communities (KW, *X*^2^ = 37.2, *p* < 0.001). Particularly, we identified that *A. andersoni* and *A. dumerilii* presented the highest values of Observed ASVs and did not differ between them (*p* = 0.36). In contrast, *A. mexicanum* and *A. taylori* had lower diversity values and did not significantly differ between them (*p* = 0.35; Fig. 6A). The same pattern was identified with fungi (Fig. 6B). Beta diversity analysis based on Bray–Curtis distances showed that skin bacterial and fungal community structure differed significantly across hosts (bacteria: Fig. 6C, PERMANOVA, Pseudo-F = 17, *p* < 0.001; and fungi: Fig. 6D, PERMANOVA, Pseudo-F = 5.3, *p* < 0.001). The percentage of variance explained was higher in bacterial communities based on the PERMANOVA models (R2 = 46%) compared to fungi (R2 = 19%). Bacterial and fungal community structure using Jaccard distances showed similar results to Bray–Curtis (Table S5). Skin microbial dispersion (for both Bary-Curtis and Jaccard distances) significantly differed among host species (Table S5).

### Host Species and Environmental Conditions Partially Explain Skin Microbial Diversity of Axolotl Microbiota

To explore environmental differences among the habitats of the four host species, we performed a principal component analysis (PCA) and a PERMANOVA using climatic and physicochemical variables (Table S8). The first two components of the PCA accounted for 83.35% of the total variance (PC1 = 62.28% and PC2 = 21.07%, Figure S5). The main variables that contributed to PC1 were mean temperature of driest quarter (Bio_9), mean temperature of wettest quarter (Bio_8), and mean temperature of warmest quarter (Bio_10), while Mean temperature of warmest quarter (Bio_14), precipitation of warmest quarter (Bio_18), and mean diurnal range (Bio_2) contributed the most to PC2. We found significant differences in environmental conditions between localities (PERMANOVA, Pseudo-F: 1835.8, *p* < 0.001).

To identify the drivers of skin microbial alpha diversity (observed ASVs), we fitted two linear models (one for bacteria and one for fungi). We found that temperature annual range (Bio_7), precipitation (Pp), and monthly minimum temperature (Tmin) had a significant effect on bacterial alpha diversity (Table S6). On the other hand, only minimum temperature of the coldest month (Bio_6) and Bio_7 had a significant effect on fungal alpha diversity. We identified that all variables had a positive relationship with skin microbial diversity, being Tmin and Bio_7 the strongest predictors (higher estimate values) of bacterial and fungal alpha diversity, respectively (Table S6). Both models predicted a significant effect of Bio_7 over skin bacterial and fungal alpha diversity. Finally, the bacterial and fungal models accounted for 70% and 42% of the observed variance (R2), respectively.

**Fig. 7:**
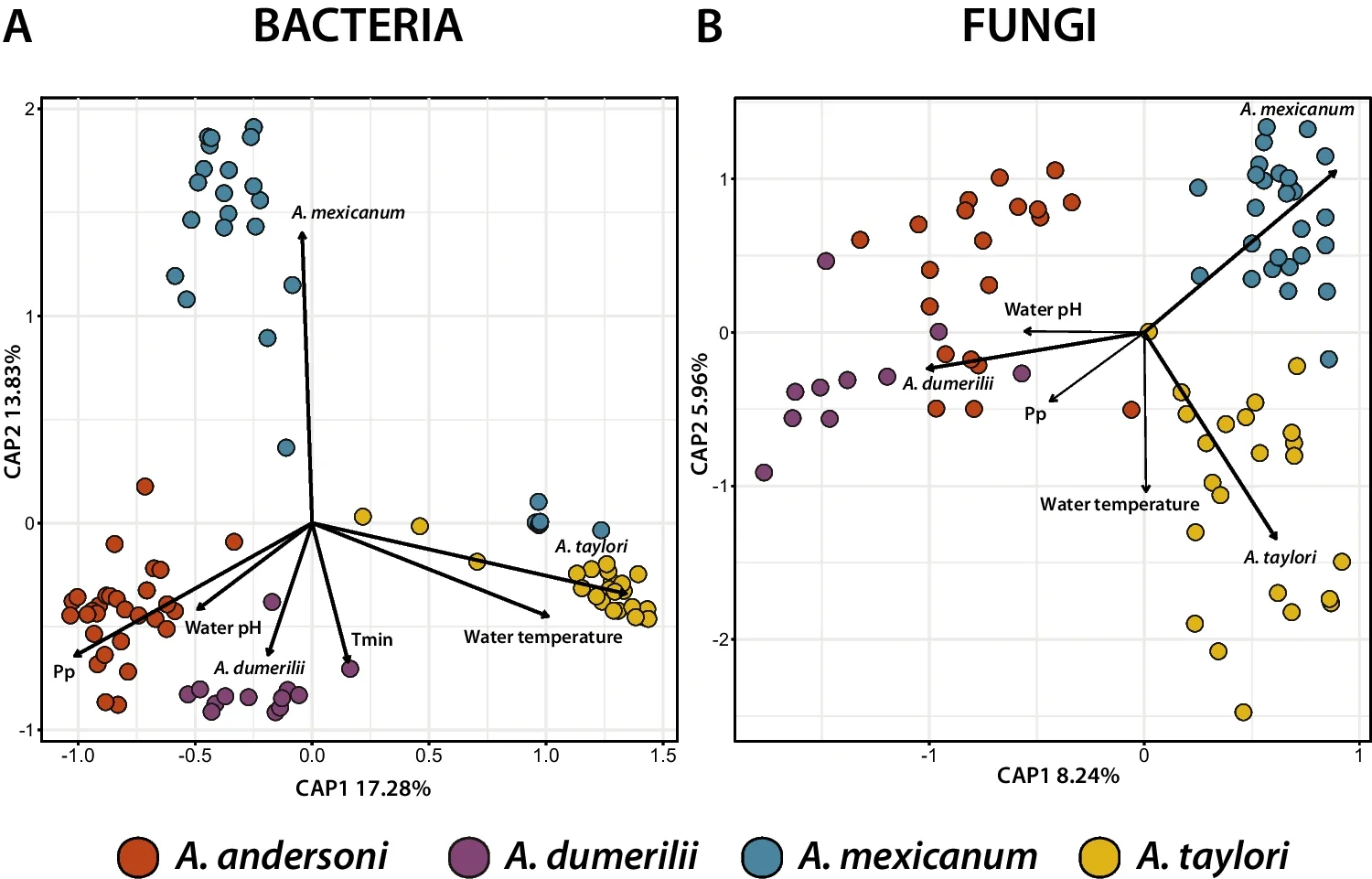
Distance-based redundancy analysis (dbRDA) of axolotl skin microbial communities. Bidimensional plots for (A) bacteria and (B) fungi. PCAs were constructed from Bray–Curtis dissimilarity distances. Vector directions indicate the type of correlation of each predictor variable. Thicker vectors indicate variables that had a significant effect. Distance of each sample with respect to vectors highlight the weight of the correlation with a given predictor variable. Circles are color-coded by host species as shown in the lower legend. See Table S1 in for variable names definitions

To identify the factors that explained skin bacterial and fungal community structure (beta diversity), we performed a distance-based redundancy analysis (dbRDA) (Fig. 7). We found that water pH, water temperature, Pp, and Tmin significantly explained variation of the bacterial community structure. In the case of the fungal communities, we did not identify a significant correlation of specific environmental variables with community structure (Table S7). Finally, based on the constrained variance of the dbRDA model, we identified that host species and environmental variables collectively explained more microbial composition variation in bacteria (R2 = 0.46) than in fungi (R2 = 0.2).

## Discussion

In this study, we characterized the bacterial and fungal skin-associated microbiota of the four obligate paedomorphic *Ambystoma* species endemic to Mexico. We found that (1) skin microbial structure is distinct from aquatic microbial communities and that (2) the majority of the microbial taxa are unique to each host species. However, (3) axolotl species shared a common set of skin bacterial and fungal taxa with high relative abundances across all hosts. Finally, (4) host species and environmental variables collectively explained more microbial composition variation in bacterial than in fungal skin-associated microbiota.

### A Unique Set of Microbes, Distinct from Environmental Communities, Defines Each Axolotl Species

Since obligate paedomorphic axolotls remain in an aquatic habitat for their entire lives [40], we asked if their skin microbiota was distinct from the aquatic microbial communities. Our findings are congruent with other studies, in which skin bacterial [11, 15, 41] or fungal [42, 43] communities differed from their environmental reservoirs. Interestingly, we identified that the structure of each axolotl skin community was more similar to their corresponding aquatic community than to the other hosts. This finding supports the idea that environmental microbial communities are the main source of microbial taxa that can eventually become associated to the skin [11, 14, 30].

In this study, we defined a set of skin-associated microbiota for each host species including not only the most prevalent ASVs in the skin samples [44], but also ASVs that were unique and significantly enriched in skin samples. With this, we analyzed the microbes that are consistently present and enriched in their host skin and are more likely to be symbionts and not just a reflection of the microbes from the environment. Our results showed that each host species harbors a unique set of ASVs, which represent most of their skin taxonomic diversity. In addition, several taxa were significantly enriched on each host species, suggesting a strong role of host-specific factors shaping their skin microbial communities. Some of these factors could be linked to the immunity and chemistry of each amphibian species, such as the content of peptides, lipids, carbohydrates, and alkaloids [45, 46]. Many of these components will likely favor the colonization of specific microbial taxa on each host species [47–49]. In light of our results, future studies should aim to describe the chemical composition of the axolotl skin.

### The Axolotl Skin Shares a Common Set of Bacterial and Fungal Taxa

Despite the unique microbial signatures associated to each host species, we found that the four axolotl species shared a common set of ASVs which had a low percentage of the total ASVs, but had overall high relative abundances. Based on previous evidence of phylosymbiosis on caudate skin bacteria [7, 50], it is interesting to find bacterial taxa that are shared and abundant across the four Ambystoma species (such as Burkholderiales and Pseudomonadales) and that are also abundant in the skin of other Caudata (salamanders) [41, 51] including other *Ambystoma* species [7, 15]. Thus, these taxa might have a long-term evolutionary relationship with Caudata species that could go back to early divergences in this amphibian order.

The mechanisms driving host species differences are not well understood, although it is likely a combination of effects given by environmental reservoirs, host-specific factors, and microbial community interactions [13, 14, 52]. In our study system, we found that skin microbial alpha and beta diversity were clearly associated to host species, but since host species identity and host habitat are confounded in this system, we cannot tease apart the host and locality effects. However, comparisons between hosts revealed that *A. andersoni* and *A. dumerilii* presented more similar microbial communities, in contrasts with *A. mexicanum* and *A. taylori*. These results are in agreement with the phylogenetic distance among these species, as shown previously [20]. Thus, host phylogenetic divergence might also play a role in driving assembly of the skin community in these salamander species [7, 53]. Nevertheless, future analyses may consider more *Ambystoma* species and additional host-associated variables to explore the influence of phylogeny and other factors such as host immune response [54], immune genetic diversity [55], or diet [56] that could be contributing to skin microbial differences.

### Host Species and Environmental Variables Explained Skin Bacterial Communities’ Variation More Than Fungal Communities

In addition to host-associated factors, local habitat environment could also be influencing skin microbiota differentiation. In this study, we showed that each location differed in local physicochemical environmental conditions as well as climate, and we were able to identify variables that partially explained the skin microbial diversity and composition variation in conjunction with the host-species effect. Interestingly, we found that host species and habitat environment had a greater effect on skin bacterial communities than in fungal communities. This might suggest that host and environmental factors may play differential roles in bacterial and fungal community assembly [57, 58].

How environmental factors impact skin microbial communities in amphibians is not well understood. In accordance with previous studies [13, 18], we found that bioclimatic variables associated to temperature and local conditions, like water pH, were correlated to bacterial diversity and composition. It is known that temperature affects the growth of bacteria and modulates the production of metabolites [59], and can also have an impact on host physiology and host-symbiotic relationships [60]. In environmental bacterial communities, pH is an important driver of diversity [61]. Also, pH variation can modulate microbial interactions, and thus, may influence the structure of microbial communities [62]. Less is known about the influence of pH on animal microbiomes, but studies in humans [63], fish [64], and amphibians [15, 37] showed that changes in pH are associated to microbial community differences. In our study, the skin fungal community structure did not seem affected by the environmental variables tested here. Thus it is plausible that other host-associated factors could be driving fungal communities, as seen in other organisms [65–67], or that other elements of the environment need to be considered. In sum, more work is needed to describe the effect of environmental and host factors in amphibian skin microbial communities (in particular fungal communities), since changes in community structure and diversity may also be linked to changes in functionality and pathogen protection [5, 68].

Our findings described the skin microbial communities in *Ambystoma* species and contributed to determine host and climatic factors that partially explain their diversity and structure. Future studies exploring host immune and genetic diversity, as well as characterization of the skin chemical environment, should further advance our understanding of host-microbiota symbiotic relationships in salamanders.

## Supplementary Information

Below is the link to the electronic supplementary material.

Supplementary file1 (DOCX 973 KB)

Supplementary file2 (XLSX 60.0 KB)

## Data Availability

The code, samples metadata and supporting data are available at https://github.com/Enrique-SC/PaedomorphicSalamanders_SkinMicrobiota. 16S and ITS raw sequence data are publicly available in NCBI under the Bioproject PRJNA924965.
