# Supplementary material for: Host Species and Environment Shape the Skin Microbiota of Mexican Axolotls
Source: Microb Ecol. 2024 Jul 24;87:98. doi: 10.1007/s00248-024-02411-1 (PMC11269437; doi:10.1007/s00248-024-02411-1)
Supplement: Supplementary file 1 [file 248_2024_2411_MOESM1_ESM.docx]

**Host Species and Environment Shape the Skin Microbiota of Mexican Axolotls.**

Enrique Soto-Cortés^1^, Montserrat Marroquín-Rodríguez^2^, Maria Delia Basanta^3,4^, Yurixhi Maldonado-López^5^, Gabriela Parra-Olea^6^, Eria A. Rebollar^1^.

1. Centro de Ciencias Genómicas, Universidad Nacional Autónoma de México, Cuernavaca, Mexico.

2. Facultad de Medicina, Universidad Nacional Autónoma de México, Mexico City, Mexico.

3. Department of Biology, University of Nevada Reno, Reno, NV, USA.

4. Facultad de Ciencias, Universidad Nacional Autónoma de México, Mexico City, Mexico.

5. Cátedras CONAHCYT - Instituto de Investigaciones sobre los Recursos Naturales, Universidad Michoacana de San Nicolás de Hidalgo, Morelia, Michoacán, Mexico.

6. Instituto de Biología, Universidad Nacional Autónoma de México, Mexico City, Mexico.

**SUPPLEMENTARY FILE 1**

1. **SUPPLEMENTARY METHODS**

**Amphibian sampling**

**Processing and sequencing of the skin bacterial and fungal microbial communities**

2. **SUPPLEMENTARY FIGURES**

**Figure S1.** Venn diagrams showing unique and shared bacterial ASVs between axolotl skin and water microbiota for each host species.

**Figure S2.** Venn diagrams showing unique and shared fungal ASVs between axolotl skin and water microbiota for each host species.

**Figure S3.** Beta diversity of axolotl skin and water microbiota for all hosts.

# Figure S4. Venn diagrams for each host species showing the skin-associated ASVs.

**Figure S5.** Principal Component Analysis of the environmental matrix data associated to four habitats where axolotls were sampled.

3. **SUPPLEMENTARY TABLES**

# Table S1. Environmental and bioclimatic variables name and definition used in the study.

# Table S2. Final bacterial and fungal sample size after the rarefaction process.

# Table S3 . PERMANOVA and PERMUTEST comparisons between skin and water samples for each species based on Bray-Curtis dissimilarity distances.

# Table S4. PERMANOVA and PERMUTEST comparisons between skin and water samples for each species based on Jaccard similarity distances.

# Table S5. PERMANOVA and PERMUTEST comparisons among host species using Bray-Curtis and Jaccard similarity distances for bacteria and fungi

**Table S6.** Lineal mixed models of alpha diversity, showing the effect of selected environmental predictors on observed ASVs in skin bacterial and fungal communities in axolotls.

**Table S7.** Permutational multivariate analysis of variance (PERMANOVA) models, showing the effect of predictors introduced on the dbRDA model regression based on Bray-Curtis dissimilarity distances for skin bacterial and fungal communities.

**SUPPLEMENTARY FILE 2**

**Table S8.** Metadata associated to each locality and host species.

**Table S9.** Taxonomy of microbial ASVs shared across host species.

- **Table S9A.** Taxonomy of the bacterial ASVs shared across host species.
- **Table S9B.** Taxonomy of the fungal ASVs shared across host species.

**Table S10.** Taxonomy of ASVs that are significantly enriched on each host species according to LEFSE analysis

- **Table S10A.** Taxonomy of bacterial ASVs that are significantly enriched on each host species according to LEFSE analysis
- **Table S10B.** Taxonomy of fungal ASVs that are significantly enriched on each host species according to LEFSE analysis

1. **SUPPLEMENTARY METHODS**

**Amphibian sampling**

To capture individuals, we used different techniques according to each species and habitat. To collect *A. andersoni* individuals, we used fish nets of 1.5 m wide per 50m long at 2-5 m from the lake shore for 12hs (19:00 – 07:00hs) and collected them the day after. We repeated this procedure 3 times across 2 months. For *A. dumerilii*, we used 180 metallic cylinder traps with a conical inlet, set at the bottom of the lake. The traps were kept at the bottom of the lake (20 m of depth) by the weight of it, for 12hs (19:00 – 07:00hs). To identify the organism collected in the lake, we use a microchip marking system, to avoid resampling. Traps were collected the day after, and this procedure was repeated 4 times across 6 months. For *A. mexicanum,* we sampled individuals from four different mesocosms for 4 hrs (11:30-15:30 hrs) using hand nets in one single day. For *A. taylori*, we dove for two consecutive nights at a depth of 12 m and captured them with a hand net. Once the individuals were captured, they were placed in individual containers with water from their habitat. Each individual was manipulated using a new pair of nitrile gloves to swab and then released at the end of sampling. All equipment (nets, traps, containers) were cleaned and disinfected before their use.

**Processing and sequencing of the skin bacterial and fungal microbial communities**

The DNA from all swabs was extracted with the Qiagen DNeasy Blood and Tissue Kit (Qiagen, Valencia, CA, USA). A pre-treatment with lysozyme was included as a first step with a lysis buffer (0.315% Tris-HCl, 0.074% EDTA, 0.12% Triton-x-100) with 20 mg lysozyme per 1 ml of lysis buffer added immediately before use.

For 16S library preparation, we used the barcoded primers (F515/R806) to amplify the V4 region of the 16S rRNA gene according to Caporaso *et al.*, (2011). PCR reactions per sample were run in triplicate with one negative control (water instead of DNA as template). PCR products and negative controls were verified in 1% agarose gels and only triplicate amplicons, with negative controls without amplification, were combined and quantified using the Qubit 2.0 Fluorometer using the dsDNA HS Assay Kit (Life Technologies). When constructing the 16S amplicon libraries, all field control samples were included but none of them amplified and thus, they were not included for sequencing. The final pool of samples was created by combining equimolar ratios of amplicons (200 ng/sample) from each sample and was cleaned with the QIAquick Qiagen PCR clean-up kit. The barcoded composite PCR products were sent to the Molecular Biology Core Facilities at Dana-Farber Cancer Institute (Boston, MA, USA) for 250PE MiSeq Illumina sequencing.

For the skin fungal microbiome, we sent 23 uL of DNA extractions to Minnesota Genomics Center (MN, USA) for ITS1 library construction using the primers ITS1F_Nextera (TCGTCGGCAGCGTCAGATGTGTATAAGAGACAGCTTGGTCATTTAGAGGAAG*TAA) and ITS1R_Nextera (GTCTCGTGGGCTCGGAGATGTGTATAAGAGACAGGCTGCGTTCTTCATCGA*TGC) and followed by 300PE MiSeq Illumina sequencing.

2. **SUPPLEMENTARY FIGURES**

Figure S1. Venn diagrams showing unique and shared bacterial ASVs between axolotl skin and water microbiota for each host species: (A) *A. andersoni,* (B) *A. dumerilii,* (C) *A. mexicanum* and (D) *A. taylori.* Parenthesis indicate the percentage of unique and shared ASVs between sample types.

# Figure S2 Venn diagrams showing unique and shared fungal ASVs between axolotl skin and water microbiota for each host species: (A) *A. andersoni,* (B) *A. dumerilii,* (C) *A. mexicanum* and (D) *A. taylori.* Parenthesis indicate the percentage of unique and shared ASVs between sample types.

# Figure S3 . Beta diversity of axolotl skin and water microbiota for all hosts. Principal Coordinates Analysis (PCoAs) of (A) bacterial and (B) fungal communities using Bray-Curtis and Jaccard distances, respectively (C and D). Shapes in legends denote host species origin. Colors in legend denote the sample type. Asterisks indicate statistically significant differences between sample types: *** = p-value < 0.001.

# Figure S4. Venn diagrams for each host species showing the skin-associated ASVs using three different criteria: 1) ASVs that were unique only to the skin samples (“Unique ASVs”), 2) ASVs present in at least 80% of skin samples, excluding ASVs that were highly prevalent in water samples (“80% criteria”), 3) ASVs significantly enriched on the skin in contrast with water samples (“Enriched ASVs”). A-D represent bacterial ASVs and E-H represent fungal ASVs for *A. andersoni*, *A. dumerilii*, *A. mexicanum* and *A. taylori,* respectively.

# Figure S5. Principal Component Analysis of the environmental matrix data associated to four habitats where axolotls were sampled: Zacapu Lake, Michoacán (red, *A. andersoni* habitat), Pátzcuaro Lake, Michoacán (yellow, *A. dumerilii* habitat), Mexico City, CDMX (green, *A. mexicanum* habitat), Alchichica Lake, Puebla (blue, *A. taylori*). Vector directions indicate the type of correlation of each predictor variable. See supporting table 1 for variable names.

3. **SUPPLEMENTARY TABLES**

# Table S1. Environmental and bioclimatic variables name and definition used in the study.

| **Variable name** | **Variable definition** |
| --- | --- |
| **SVL** | Snout vent length (cm) of each individual |
| **Weight** | Weight (g) of each individual |
| **Elevation** | The height of a geographic location above mean sea level |
| **Water temperature** | Mean water temperature of the lake where the axolotls were sampled |
| **Water pH** | Mean water pH of the lake where the axolotls were sampled |
| **Pp** | Mean value of monthly precipitation across 2018 |
| **Tmin** | Mean value of monthly minimum temperature across 2018 |
| **Tmax** | Mean value of monthly maximum temperature across 2018 |
| **DO** | Mean dissolved oxygen values in the lake water where the axolotls were sampled |
| **BIO_1** | Annual Mean Temperature |
| **BIO_2** | Mean Diurnal Range (Mean of monthly (max temp - min temp)) |
| **BIO_3** | Isothermality (BIO2/BIO7) (×100) |
| **BIO_4** | Temperature Seasonality (standard deviation ×100) |
| **BIO_5** | Max Temperature of Warmest Month |
| **BIO_6** | Min Temperature of Coldest Month |
| **BIO_7** | Temperature Annual Range (BIO5-BIO6) |
| **BIO_8** | Mean Temperature of Wettest Quarter |
| **BIO_9** | Mean Temperature of Driest Quarter |
| **BIO_10** | Mean Temperature of Warmest Quarter |
| **BIO_11** | Mean Temperature of Coldest Quarter |
| **BIO_12** | Annual Precipitation |
| **BIO_13** | Precipitation of Wettest Month |
| **BIO_14** | Precipitation of Driest Month |
| **BIO_15** | Precipitation Seasonality (Coefficient of Variation) |
| **BIO_16** | Precipitation of Wettest Quarter |
| **BIO_17** | Precipitation of Driest Quarter |
| **BIO_18** | Precipitation of Warmest Quarter |
| **BIO_19** | Precipitation of Coldest Quarter |

# Table S2. Final bacterial and fungal sample size after the rarefaction process.

| **Kingdom** | **Host species** | **Samples** | | |
| --- | --- | --- | --- | --- |
|  |  | **Skin** | **Water** | **Total** |
| **BACTERIA** | *A. andersoni* | 30 | 4 | 34 |
|  | *A. dumerilii* | 13 | 2 | 15 |
|  | *A. mexicanum* | 26 | 15 | 41 |
|  | *A. taylori* | 23 | 5 | 28 |
|  | **Total samples** | **92** | **26** | **118** |
| **FUNGI** | *A. andersoni* | 20 | 3 | 23 |
|  | *A. dumerilii* | 10 | 2 | 12 |
|  | *A. mexicanum* | 27 | 15 | 42 |
|  | *A. taylori* | 23 | 5 | 28 |
|  | **Total samples** | **80** | **25** | **105** |

# Table S3 . PERMANOVA and PERMUTEST comparisons between skin and water samples for each species based on Bray-Curtis dissimilarity distances. Numbers in bold indicate statistically significant p-values of each comparison.

| **Kingdom** | **Sample type comparison (axolotl skin vs water)** |  | | **PERMANOVA** | | | | **PERMUTEST** | |
| --- | --- | --- | --- | --- | --- | --- | --- | --- | --- |
|  |  | **DF** | **Residuals** | | **Pseudo-F** | **R2** | **p-value** | **F** | **p-value** |
|  | All samples | 1 | 117 | | 10.32 | 0.0817 | **< 0.001** | 1.49 | 0.214 |
| **BACTERIA** | *A. andersoni* | 1 | 31 | | 4.51 | 0.1236 | **< 0.001** | 8.281 | **< 0.001** |
|  | *A. dumerilii* | 1 | 13 | | 6.85 | 0.34523 | **< 0.01** | 3.73 | 0.128 |
|  | *A. mexicanum* | 1 | 39 | | 14.9 | 0.2765 | **< 0.001** | 5.9 | **0.022** |
|  | *A. taylori* | 1 | 26 | | 7.28 | 0.2186 | **< 0.001** | 4.13 | 0.052 |
|  | All samples | 1 | 103 | | 4.69 | 0.0435 | **< 0.001** | 0.11 | 0.729 |
| **FUNGI** | *A. andersoni* | 1 | 21 | | 1.48 | 0.0658 | 0.095 | 1.23 | 0.219 |
|  | *A. dumerilii* | 1 | 10 | | 1.85 | 0.15672 | **0.024** | 0.012 | 0.871 |
|  | *A. mexicanum* | 1 | 40 | | 7.48 | 0.15752 | **< 0.001** | 2.335 | 0.145 |
|  | *A. taylori* | 1 | 26 | | 1.54 | 0.05613 | **0.031** | 4.52 | **0.039** |

# Table S4. PERMANOVA and PERMUTEST comparisons between skin and water samples for each species based on Jaccard similarity distances.

| **Kingdom** | **Sample type comparison (axolotl skin vs water)** |  | | **PERMANOVA** | | | | **PERMUTEST** | |
| --- | --- | --- | --- | --- | --- | --- | --- | --- | --- |
|  |  | **DF** | **Residuals** | | **Pseudo-F** | **R2** | ***p-value*** | **F** | ***p-value*** |
|  | All samples | 1 | 117 | | 4.37 | 0.0363 | **< 0.001** | 27.39 | **< 0.001** |
| **BACTERIA** | *A. andersoni* | 1 | 31 | | 1.87 | 0.057 | **< 0.001** | 118.9 | **< 0.001** |
|  | *A. dumerilii* | 1 | 13 | | 1.91 | 0.128 | **0.01** | 54.3 | **< 0.001** |
|  | *A. mexicanum* | 1 | 39 | | 5.37 | 0.121 | **< 0.001** | 69.6 | **< 0.001** |
|  | *A. taylori* | 1 | 26 | | 2.42 | 0.085 | **< 0.001** | 20 | **0.002** |
|  | All samples | 1 | 103 | | 1.95 | 0.018 | **< 0.001** | 12.7 | **< 0.001** |
| **FUNGI** | *A. andersoni* | 1 | 21 | | 1.24 | 0.056 | **0.026** | 46.8 | **< 0.001** |
|  | *A. dumerilii* | 1 | 10 | | 1.51 | 0.131 | **0.018** | 82.2 | **< 0.001** |
|  | *A. mexicanum* | 1 | 40 | | 1.82 | 0.043 | **< 0.001** | 19.5 | **< 0.001** |
|  | *A. taylori* | 1 | 26 | | 1.16 | 0.042 | **0.014** | 24.8 | **< 0.001** |

# Table S5. PERMANOVA and PERMUTEST comparisons among host species using Bray-Curtis and Jaccard similarity distances for bacteria and fungi. Numbers in bold indicate statistically significant p-values of each comparison.

| **Kingdom** | **Matrix distance** |  | | | **PERMANOVA** | | | **PERMUTEST** | |
| --- | --- | --- | --- | --- | --- | --- | --- | --- | --- |
|  |  | **DF** | **Residuals** | **Pseudo-F** | | **R2** | **p-value** | **F** | ***p-value*** |
| **BACTERIA** | Bray-Curtis | 3 | 87 | 16.9 | | 0.3683 | **< 0.001** | 4.68 | **0.006** |
|  | Jaccard | 3 | 87 | 9.2 | | 0.242 | **< 0.001** | 10.17 | **< 0.001** |
| **FUNGI** | Bray-Curtis | 3 | 76 | 5.3 | | 0.1731 | **< 0.001** | 15.75 | **< 0.001** |
|  | Jaccard | 3 | 76 | 3.3 | | 0.116 | **< 0.001** | 29.28 | **< 0.001** |

**Table S6.** Lineal mixed models of alpha diversity, showing the effect of selected environmental predictors on observed ASVs in skin bacterial and fungal communities in axolotls. See Table S1 for variable names definition. Numbers in bold indicate statistically significant p-values.

| **Kingdom** | **Variables** | **Estimate** | **Std.Error** | **t-value** | ***p-value*** |
| --- | --- | --- | --- | --- | --- |
| **BACTERIA** | (Intercept) | 189.32967 | 6.72708819 | 28.1443717 | **< 0.001** |
|  | Bio_7 | 38.0695418 | 7.16094693 | 5.31627202 | **< 0.001** |
|  | Pp | 25.4145776 | 8.26344003 | 3.07554451 | **< 0.01** |
|  | Tmin | 69.5098657 | 8.46520941 | 8.21123995 | **< 0.001** |
| **FUNGI** | (Intercept) | 66.175 | 3.68017837 | 17.9814654 | **< 0.001** |
|  | Water temperature | 7.59086839 | 4.53417233 | 1.67414642 | 0.09 |
|  | Bio_6 | 33.6138531 | 5.28807528 | 6.35653831 | **< 0.001** |
|  | Bio_7 | 31.4595823 | 4.70065294 | 6.69259839 | **< 0.001** |
|  | Pp | 6.99521 | 3.74554585 | 1.86760916 | 0.06 |

# Table S7. Permutational multivariate analysis of variance (PERMANOVA) models, showing the effect of predictors introduced on the dbRDA model regression based on Bray-Curtis dissimilarity distances for skin bacterial and fungal communities. Numbers in bold indicate statistically significant p-values. See Table S1 for variable names definition.

| **Kingdom** | **Variables** | **Df** | **Residuals** | **Pseudo-F** | ***p-*value** |
| --- | --- | --- | --- | --- | --- |
| **BACTERIA** | Host species | 3 | 83 | 18.4 | **< 0.001** |
|  | Water pH | 1 | 83 | 4.05 | **< 0.001** |
|  | Water temperature | 1 | 83 | 3 | **< 0.01** |
|  | Pp | 1 | 83 | 3.2 | **< 0.01** |
|  | Tmin | 1 | 83 | 4.5 | **< 0.001** |
| **FUNGI** | Host species | 3 | 74 | 5.2 | **< 0.001** |
|  | Water temperature | 1 | 74 | 1.1 | < 0.25 |
|  | Water pH | 1 | 74 | 0.76 | 0.9 |
|  | Pp | 1 | 74 | 1.05 | 0.34 |
